# Efficacy of a Ready-to-Feed Starter Liquid Infant Formula Containing 2-Fucosyllactose and Lacto-N-Neotetraose in Chinese Infants: Protocol for a Double Blind, Randomized Controlled Trial

**DOI:** 10.2196/66489

**Published:** 2025-10-31

**Authors:** Ying Wang, Caroline Ivanne Le Roy, Jing Li, Shuping Han, Zhiwei Liu, Anirban Lahiry, Himanshu Sahu, Qiaoji Li, Jie Dong, Alric Mondragon, Tinu Mary Samuel, Wei Cai

**Affiliations:** 1 Division of Pediatric Gastroenterology and Nutrition Xinhua Hospital School of Medicine, Shanghai Jiao Tong University Shanghai China; 2 Clinical Research Unit Société des Produits Nestlé SA 1000 Lausanne Switzerland; 3 Shanghai First Maternity and Infant Hospital School of Medicine Tongji University Shanghai China; 4 Women's Hospital of Nanjing Medical University Nanjing Women and Children’s Healthcare Hospital Nanjing China; 5 The International Peace Maternity and Child Health Hospital of China Welfare Institute Shanghai China; 6 Clinical Research Unit Nestlé Research Beijing China; 7 Wyeth Nutrition Shanghai China; 8 Nestlé Product Technology Center-Nutrition Société des Produits Nestlé SA Vevey Switzerland; 9 Shanghai Institute for Pediatric Research Shanghai China

**Keywords:** Bifidobacteria, gut microbiome, gastrointestinal tolerance, growth, immune health, human milk oligosaccharides, infant nutrition

## Abstract

**Background:**

Bioactive compounds, such as human milk oligosaccharides (HMOs), impact the development of the intestinal microbiome and immune maturation in early life. They have been shown to result in positive benefits, including improved gut health, reduced frequency of infections, and age-appropriate growth when added to infant formula. However, data supporting the added value of including these HMOs in early-stage infant formula is currently lacking among Chinese infants.

**Objective:**

In this double-blind randomized controlled trial including a nonrandomized reference breastfed group, we will test the efficacy of ready-to-feed infant formula containing 2 HMOs (2-fucosyllactose and lacto-N-neotetraose) on *Bifidobacteria* abundance, gut microbiome, gut and immune health, growth, and quality of life.

**Methods:**

Healthy infants will be enrolled between 3 and 14 days after birth at 5 sites in China and randomized to either the experimental group (fed ready-to-feed infant formula containing 2 HMOs) or the control group (fed the same ready-to-feed infant formula without the 2 HMOs), using a dynamic allocation algorithm with double blinding. Infants will receive trial formula until age 6 months and will be followed up until age 12 months. The breastfed group will serve as a reference. The primary end point will be *Bifidobacteria* abundance in fecal samples at 3 months, measured via quantitative polymerase chain reaction. Secondary end points will include fecal microbiome (including taxonomy, diversity, functionality, and metabolites), fecal markers of immune health, gastrointestinal tolerance, stooling patterns, immune competence (overall state of the immune system), sleep quality, growth, quality of life, medication use, and physician-reported adverse events. A 2-sided test at the 5% significance level will be used for statistical testing.

**Results:**

The study received ethical approval in March 2024 and will be completed by the end of 2026, which will be followed by a publication in a peer-reviewed journal.

**Conclusions:**

The Starter Liquid Infant Formula Trial (STARLIT) will be one of the first to assess the efficacy of these 2 HMOs among Chinese infants on gut and immune health, in addition to clinically relevant outcomes such as quality of life, growth, and adverse events. This study should help to demonstrate that an increase in the growth of beneficial *Bifidobacteria* in response to intake of 2-fucosyllactose and lacto-N-neotetraose may have a broader impact on overall gut microbiome composition and infant gut and immune health.

**Trial Registration:**

ClinicalTrials.gov NCT06361719; https://clinicaltrials.gov/study/NCT06361719

**International Registered Report Identifier (IRRID):**

PRR1-10.2196/66489

## Introduction

Human milk provides an abundance of structurally diverse bioactive compounds that promote appropriate gut maturation through their effects on the gut microbiome, gut barrier function, and intestinal immunity [1-8]. These bioactive compounds include human milk oligosaccharides (HMOs), α-lactalbumin, osteopontin, lactoferrin, gangliosides, phospholipids, immunoglobulins, and sphingomyelin [5-7,9]. Specifically, HMOs play a crucial role in the development of the intestinal microbiome and immune maturation [10,11] as they promote the colonization of beneficial bacteria such as *Bifidobacteria* [12,13] and improve gut epithelial barrier function by preventing the uptake of pathogens and toxins in the gut [13]. More specifically, 2-fucosyllactose and lacto-N-neotetraose have been found to enhance fermentation by gut microbes [14] and have antibacterial [15,16], antiviral [17,18], and anti-inflammatory activities [17] in vitro. In preclinical models, 2-fucosyllactose and lacto-N-neotetraose enhance fermentation and short-chain fatty acids production [18] by the gut microbiome [14] by promoting the growth of the beneficial *Bifidobacterium* species [15-19], while inhibiting colonization by pathogenic bacteria [18-20]. Preclinical evidence also suggests that 2-fucosyllactose and lacto-N-neotetraose exhibit antibacterial action beyond the gut, specifically the upper respiratory tract [21,22], as well as demonstrating antiviral [23,24] and anti-inflammatory activity [19,20,24].

Term infant formula supplemented with up to 1.0 g/L of 2-fucosyllactose alone [25,26] or in combination with 0.5 g/L of lacto-N-neotetraose [27] is well tolerated and supports normal growth. Infants fed formula supplemented with 1.0 g/L 2-fucosyllactose and 0.5 g/L of lacto-N-neotetraose in the first 6 months of life had lower parent-reported morbidity, including bronchitis and lower respiratory tract infections (LRTI) through the first 12 months of life compared to infants fed the same cow’s milk–based infant formula without HMOs [27]. Additionally, lower antipyretics at age 4 months and antibiotics at ages 6 and 12 months were recorded in the HMO-fed group [27].

Part of the observed benefits of 2-fucosyllactose and lacto-N-neotetraose consumption in early life may be mediated by the effects of these 2 HMOs on the maturing gut microbiome. Consumption of 2-fucosyllactose and lacto-N-neotetraose containing formula in early life was associated with higher abundance of fecal *Bifidobacteriaceae* compared to non-HMO containing formula [28] and a gut microbiome composition closer to that of the breastfed group (BG), characterized by a greater likelihood to have a fecal community type similar to the most predominant of breastfed infants (defined by a high abundance of *Bifidobacteriaceae*). The latter was associated with lower antibiotic usage [28] and a distinct gut microbiome signature associated with reduced incidence of LRTI [29]. Finally, extensively hydrolyzed whey–based formula supplemented with these HMOs (2-fucosyllactose and lacto-N-neotetraose) was also demonstrated to support normal growth, have good gastrointestinal tolerance [30], reduce the frequency of upper respiratory tract and ear infections [30], increase fecal *Bifidobacterium*, and delay shift to an adult-type microbiome in infants with cow’s milk protein allergy [31].

These findings support the rationale for supplementing cow’s milk–based infant formulas with HMOs, including 2-fucosyllactose and lacto-N-neotetraose, for use in infants where either breastfeeding or the provision of breastmilk is not possible. However, data are currently lacking in Chinese infants as to how these HMOs can positively impact them very early in life. More specifically, clinical substantiation on the impact of these 2 HMOs on the dynamic changes in gut microbiome, gut barrier integrity, immune response, and other developmental outcomes is warranted in this population.

We aim to conduct a double-blind randomized controlled trial including a nonrandomized, reference BG to assess the efficacy of a ready-to-feed, liquid, bovine milk–based whey, predominant term infant formula containing 2-fucosyllactose and lacto-N-neotetraose from the first 2 weeks of age up to age 6 months in Chinese infants. We will also study its effects postintervention for up to age 12 months in promoting appropriate gut maturation and supporting immune functions. The primary objective of this trial is to show an increase in *Bifidobacteria* abundance in the experimental group (EG, which will receive ready-to-feed infant formula containing 2 HMOs) compared to the control group (CG, which will receive the same ready-to-feed infant formula without the 2 HMOs) at age 3 months. Secondary objectives include comparing the following outcomes among all the feeding groups (EG, CG, and BG): fecal microbiome and metabolic profile, fecal markers of immune health, gut barrier integrity, and inflammation, gastrointestinal tolerance, adverse events (AEs), infant immune competence, infant sleep quality, infant growth, and infant quality of life from enrollment up to age 12 months.

## Methods

### Trial Design

**Figure 1:**
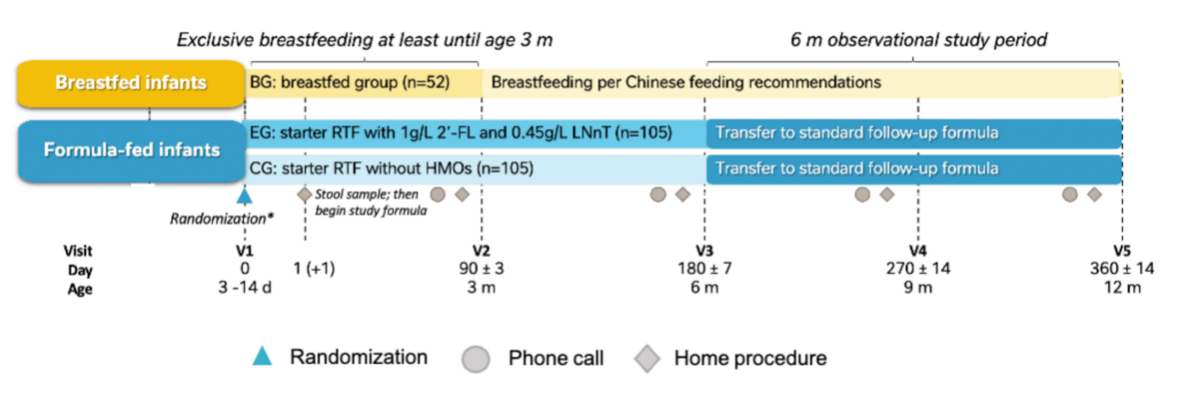
Graphical representation of the 3-arm study design. Randomization is for the formula-fed group only. 2’FL: 2-fucosyllactose; BG: breastfed group; CG: control group; EG: experimental group; HMO: human milk oligosaccharide; LNnT: lacto-N-neotetraose; RTF: ready-to-feed; V: visit.

This is a double-blind, randomized controlled intervention trial of healthy male and female term infants. Briefly, the trial will include three parallel arms: (1) the EG that will consume the interventional product (ready-to-feed infant formula containing 2 HMOs), (2) the CG that will consume the comparator product (CP, ready-to-feed infant formula without the 2 HMOs), and a BG as reference. The study constitutes of an intervention period (age 0-6 months) where infants from both the CG and EG will be exposed to either the interventional product or CP to evaluate their effects on key study outcomes, followed by an observational follow-up period (age 6-12 months) to evaluate long term postintervention effects of interventional product and CP on gut microbiome maturation and later incidence of illnesses, infections as well as immune status (Figure 1). The trial will include 5 scheduled clinic visits (V1: infant age 3-14 days, V2: infant age 3 months, V3: infant age 6 months, V4: infant age 9 months, and V5: infant age 12 months). Phone call appointments will serve as reminder calls for the collection of stool samples and completion of relevant questionnaires that need to be completed as part of the home procedures before the clinic visits. A brief overview of the study sessions and procedures is summarized in Table 1 and described in detail.

**Table 1:** Study sessions and procedure.

Procedures			Intervention period													Observation period						
			V1^a^		H1^b^		H2		V2		H3		V3		H4			V4		H5		V5
Informed consent			✓																			
Inclusion and exclusion criteria			✓																			
Physical examination			✓																			✓
Medical history			✓																			
Demographics			✓																			
Anthropometry			✓						✓				✓					✓				✓
Randomization			✓																			
Fecal samples					✓		✓				✓				✓					✓		
**Questionnaires**																						
	IGSQ-13^c^	✓						✓				✓										
	PISI^d^							✓				✓					✓				✓	
	BISQ^e^	✓						✓				✓										
	ITQoL-SF^f^ 47							✓				✓										
**Diaries**																						
	Stooling	✓				✓				✓												
	3-day food									✓				✓					✓			
	Breastfeeding (BG^g^ only)	✓						✓				✓					✓				✓	
	Formula milk intake	✓						✓				✓					✓				✓	
	Infant illness	✓		✓		✓		✓		✓		✓		✓			✓		✓		✓	
**Investigational product**																						
	Administer trial formula (EG^h^/CG^i^ only)			✓		✓		✓		✓		✓										
	Adverse event collection	✓		✓		✓		✓		✓		✓		✓			✓		✓		✓	

^a^V: visit.

^b^H: home.

^c^IGSQ: Infant Gastrointestinal Symptom Questionnaire.

^d^PISI: Pediatric Immune System Index.

^e^BISQ: Brief Infant Sleep Questionnaire.

^f^ITQoL-SF: Infant Toddler Quality of Life Questionnaire-Short Form.

^g^BG: breastfed group.

^h^EG: experimental group.

^i^CG: control group.

### Trial Population and Eligibility Criteria

The trial population will consist of healthy infants aged 3-14 days at enrollment. Potential participants will be referred by the clinical care team to the investigator to check whether they meet the eligibility criteria, and the investigator will provide a thorough explanation of the trial. The eligibility of the potential participant will be confirmed according to the inclusion and exclusion criteria (Textbox 1), and each participant will be assigned a unique identification number.

**Inclusion and exclusion criteria.**

**Inclusion criteria**

- Singleton, healthy, full-term gestational birth (≥37 completed weeks of gestation), with a birth weight ≥2.5 kg and ≤4.5 kg and postnatal age of 3-14 days (date of birth=day 0), inclusive.
- Willing and able to comply with scheduled visits and the requirements of this trial protocol.
- Access to a working freezer.
- For the formula-fed groups: infant is exclusively consuming and tolerating a cow’s milk infant formula for at least 3 days before enrollment. The infant’s mother has independently elected not to breastfeed.
- For the breastfed reference group: the infant has been exclusively consuming breastmilk since birth, and the infant’s mother has decided to continue until at least age 3 months.

**Exclusion criteria**

- Infants receiving complementary foods or liquids, defined as 4 or more teaspoons per day or approximately 20 g per day of complementary foods or liquids at or before enrollment.
- Infants who have a medical condition or history that could increase the risk associated with trial participation or interfere with the interpretation of trial results.
- Infants who are presently receiving or have received before enrollment probiotic supplements or medications or supplements known or suspected to affect fat digestion, absorption, or metabolism (eg, pancreatic enzymes); stool characteristics or microbiome (eg, oral and systemic antibiotics, glycerin suppositories, bismuth-containing medications, docusate, Maltsupex, or lactulose); growth (eg, insulin or growth hormone); or gastric acid secretion.
- Infants currently participating or having participated in another clinical trial since birth.
- Infants with a known or suspected cow’s milk protein intolerance or allergy, lactose intolerance, soy intolerance, or allergy.

### Study Sites and Recruitment

Infants for this trial will be recruited from the following sites in China, including Xinhua Hospital affiliated to Shanghai Jiao Tong University School of Medicine, The International Peace Maternity & Child Health Hospital of China Welfare Institute at Shanghai, Shanghai First Maternal, Infant Hospital and Nanjing Women and Children Healthcare Center, and Peking University Third Hospital. The leading site is Xinhua Hospital affiliated to Shanghai Jiao Tong University School of Medicine, which is one of the top Pediatric Research centers in China specialized and well recognized in pediatric education, health care and research, while all the other participating hospitals are among the top women-and-children’s health care hospitals, providing comprehensive health care in the north east and middle east regions of China.

At recruitment, the infants’ parents or legally authorized representative (LAR) should have already made the decision regarding the feeding choice for their infant, and only when the parents have independently decided to formula-feed their infant, the infant will be enrolled into the clinical trial. No public or commercial advertisements of the trial, including research labels or product brand names, will be used. The benefits of breastfeeding will be discussed with all parents. Breastfeeding support via appointment with a qualified lactation consultant will be provided, before enrollment, to both the breast-fed and formula-fed groups, to ensure parents have received the appropriate advice. During the study, there will be dedicated investigational staff keeping close contact with the participants to acquire knowledge of any untoward medical event of their infants, and to remind them of study-related procedures such as stool sample collection and questionnaire completion.

### Randomization Procedure and Blinding

Participants will be assigned to the EG or CG groups randomly using Medidata Randomization Trial Supply Management with the dynamic allocation algorithm at the first visit. An allocation ratio of 1:1 stratified by site, sex (female or male), and mode of delivery (cesarean section [CS] or vaginal) will be carried out. Neither the investigator or site team nor the participant will know which investigational product each participant is receiving until after database lock. Considering the double-blinded nature of the trial, the identity of the specific product will be blinded to participants, participants’ parents, or caregivers (if applicable), support staff, investigators, site team, contract research organization managing the trial (except specified delegated regulatory staff), and study sponsors (except the manufacturing site, supply, and quality managers). The products will be identical in packaging, color, and taste. They will be packaged and labeled identically but will have distinguishable product codes printed on the packaging. Unblinding will be done only in case of emergency or after database lock for final statistical analyses.

### Intervention

The EG will receive a ready-to-feed, liquid, bovine milk–based whey, predominant term formula supplemented with 1 g/L 2-fucosyllactose and 0.45 g/L lacto-N-neotetraose. The dosage of the HMO blend used in the trial is well within the range of HMO concentrations found in mature breast milk [32,33]. The formula contains 67 kcal/100 mL, 2 g protein/100 kcal (65% whey/35% casein), 10.6 g carbohydrates/100 kcal, and 5.4 g lipids/100 kcal. The CG will receive the same formula but without the HMOs. Both formulas are enriched with an advanced whey protein profile obtained using a unique whey protein ingredient that delivers a formula enriched in α-lactalbumin, osteopontin, lactoferrin, and milk-fat-globule membrane (MFGM) proteins and lipids (α-MFGM) [34] and a casein profile comprised of A2 β-Casein, in combination with an Sn-2 palmitate enriched triacylglycerol core and contain vitamins and minerals in amounts intended for full nutritional support of infants from birth to 6 months. Formulas will be started after the baseline stool sample collection on study day 1 or 2 and continue until age 6 months, after which infants will switch to a classical nontrial follow-up formula (not provided by the sponsor) until age 12 months. The study formulas will be provided in bottles of 70 mL stage 1 liquid ready-to-feed with each dispensing unit (carton) coded with a unique identifier and containing 32 bottles. The packaging and labeling of each bottle or carton will be identical for both groups, except for this unique identifier. The amount of formula offered to the infant will be informed by a physician or the recommended feeding table for this age group. Formulas will be consumed daily, ad libitum, per standard practice. The BG will continue exclusively breastfeeding at least until age 3 months and continue to breastfeed as per standard Chinese feeding recommendations up to study end.

Participants may be discontinued from the trial or the product if they withdraw consent to participate or from the trial product. This should have no impact on the participant and will be done without reprisal as per the Declaration of Helsinki. The investigator may also decide to discontinue a participant from the trial or the product if, in his or her opinion, continuation may be detrimental to the participant. Reason for discontinuation will be documented throughout the study.

### Study Outcomes

The primary end point of this trial will be *Bifidobacteria* abundance in fecal samples at age 3 months (before V2). *Bifidobacteria* play a central role in gut microbiome maturation at early stages of life. *Bifidobacteria* are the most predominant taxon during the first 6 months of life, hence we aimed to evaluate how the intervention will impact this taxon halfway through the period in which it is playing a key role in gut microbiome and immune maturation [35,36]. Secondary end points will include fecal microbiome and metabolic profile, fecal markers of immune health, gut barrier integrity and inflammation, gastrointestinal tolerance, immune competence, sleep quality, infant health-related quality of life, growth, infant feeding and dietary patterns, and AEs.

### Bifidobacteria Abundance

DNA will be extracted from the fecal samples, and microbiome composition will be assessed using next-generation sequencing (NGS) and quantitative polymerase chain reaction. The fecal sample collected at age 3 months (just before V2) will be used to examine the differences between intervention groups (EG and CG) in *Bifidobacteria* abundance, the primary end point.

### Secondary Fecal Outcomes

Fecal microbiome, including overall taxonomic and functional composition and diversity, will be assessed at all time points (V1 to V5) using NGS. The assessment includes a relative abundance of beneficial bacterial species (eg, *B infantis*), pathogenic bacterial species, α diversity, β diversity, fecal community types, and functional annotation of the collected samples. Fecal metabolic content will be measured by quantifying pH and organic acids (such as but not restricted to lactate (including indole-lactate, L- and D-lactate), propionate, butyrate, acetate, valerate, total organic acids, and total short-chain fatty acids), at all time points (V1-V5). These organic acids will be profiled using ultra-high performance liquid chromatography coupled to tandem mass spectrometry. Additionally, targeted or untargeted metabolomics will be performed using high-performance liquid chromatography on the same samples. Fecal markers of immune health (secretory immunoglobulin A), gut barrier integrity (α1-antitrypsin, calprotectin, and lipocalin-2), and inflammation (IL-1β, IFN-γ, and TNF-α) will be measured at V1, V2, and V3 and quantified using an enzyme-linked immunosorbent assay. All fecal metabolites and markers will be reported as per wet stool weight and per dry stool weight (dry stool weight will be calculated to normalize the data for analysis). Plans for collection, laboratory evaluation, and storage of fecal samples are described in further detail in Additional File S1 in Multimedia Appendix 1.

### Gastrointestinal Tolerance and Stooling Patterns

Gastrointestinal symptoms and related behaviors will be captured by the Infant Gastrointestinal Symptom Questionnaire-13 [37] at V1, V2, and V3. This is a validated, 13-item instrument of behaviors that the infant has experienced from the parent’s perspective, with 5 domains including stooling, flatulence, fussiness, crying, and vomiting. The possible range of scores for the composite index is 13-65, with lower scores indicating lower discomfort. Good tolerance, some gastrointestinal distress, and not well-tolerated are reflected by scores of 13-23, 23-30, and >30, respectively.

Stool patterns, including stool frequency, consistency, and difficulty in passing stool, will be collected using a 1-day retrospective diary at baseline (V1) and a 3-day prospective stool diary completed at home by the parents at V2 and V3. Stool frequency will be computed as the mean number of reported stools. Stool consistency will be validated using a 5-point stool scale: 1=watery, 2=runny, 3=mushy or soft, 4=firm, and 5=hard [38], and the mean scores will be calculated. Difficulty in passing stool will be calculated as the number of stools reported as difficult of the total number of stools.

### Immune Competence

The Pediatric Immune System Index is a short questionnaire developed internally for use in healthy infants to assess their overall state of the immune system and is assessed at V2 to V5. The questionnaire has 21 items across the 4 domains of gastrointestinal, upper respiratory tract infections, LRTI, and skin allergies, and records the duration, episodes, severity of health care visits, and treatment received for the most severe case. The responses recorded for each of the items are mapped to a score of 0, 1, 2, or 3. The final score (varying from 0 to 63) corresponding to a participant for a visit is then calculated as the sum of the individual item scores, with 0 indicating the best and 63 indicating the worst immune health.

### Sleep Quality

Sleep quality will be assessed at V1, V2, and V3 using the Brief Infant Sleep Questionnaire that has been validated against actigraphy and sleep diaries [39]. The Brief Infant Sleep Questionnaire is a 13-item parent-reported questionnaire on infant or toddler sleep over the past week, takes about 5-10 minutes to complete by the parent or caregiver, and assesses nocturnal sleep duration, night wakings, and method of falling asleep.

### Quality of Life

Infant quality of life will be measured at V2 and V3 using the Infant Toddler Quality of Life Questionnaire-Short Form 47, which has been validated in China [40,41]. It is a 47-item tool that asks about infant life across 9 domains: general health, physical functioning, growth and development, discomfort and pain, temperament and mood, behavior, social interaction, health perception, and anxiety and worry. Scores range from 0 to 100 for each domain, with 0 indicating the worst quality of life and 100 indicating the best quality of life.

### Growth

Anthropometric measurements, including weight (g), length (cm), and head circumference (cm), will be collected at all visits. Infants will be weighed without clothing or a diaper on a calibrated electronic weighing scale, and the weight will be recorded twice to the nearest 10 g. Using a standardized infant length board, length will be recorded twice to the nearest 0.1 cm with the infant looking vertically upward and the crown of the infant’s head in contact with the headpiece of the board. Infant head circumference will be measured twice to the nearest 0.1 cm using a standard nonelastic plastic-coated measuring tape. Sex- and age-specific *z* scores, including weight-for-age, weight-for-length, length-for-age, and head-circumference-for-age, will be calculated using the World Health Organization (WHO) Child Growth Standards as the reference population [42].

### Safety Reporting

Safety assessments will involve the monitoring and recording of all AEs and serious AEs throughout the trial upon signature of informed consent until the end of the trial or follow-up. Additional safety evaluations may be performed when medically indicated or in the opinion of the investigator. Parents or caregivers will be responsible for completing the infant illness diary (IID) weekly from enrollment until V5. The diary will ask the parent to capture the number of occurrences and the length of time the infant experiences the following symptoms: (1) fever, (2) respiratory tract symptoms, (3) gastrointestinal symptoms, (4) ear symptoms, as well as any medications the infant may be taking. The diary entries will be reviewed at each scheduled site visit or via regular phone calls, and the trial physician will validate the symptoms and associated diagnosis. Physical examinations will be performed at the baseline visit (V1) and the end (V5) or if deemed necessary at other visits for medically significant reasons. Details about the events including participant and date, description, reporting source, dispensed investigational product, duration, frequency, intensity (mild, moderate, or severe), seriousness, action taken, outcome, and sequelae if any, and relationship to test product (unrelated, unlikely, probable, or related), will be documented thoroughly.

### Additional Variables of Interest

#### Demographic Data, Birth, and Household Characteristics

Demographic data including age of child, sex, ethnicity, birth characteristics (birth weight and length, gestational age at birth, parity), APGAR (appearance, pulse, grimace, activity, and respiration) score at 5 minutes, feeding history (eg, breastmilk, formula, or complementary feedings), maternal age, education, health history and family history of allergy will be collected from the participant or parents or LARs at the enrollment visit (V1).

#### Feeding and Dietary Patterns

A 3-day food intake diary will be collected before each fecal sample is collected when the infants are aged 6, 9, and 12 months to evaluate the energy and nutrient intake, dietary pattern, and the introduction of complementary foods. Compliance with breastfeeding will be assessed among the BG infants using the breastfeeding diary, which will capture details about the frequency, duration, and type of breastfeeding (exclusive, predominant, or partial) at each visit (V1-V5). Additionally, parents or caregivers will complete the formula milk intake diary, which will collect information regarding the total quantity of milk consumed for each formula feeding from V1 to V5 in the EG and CG infants. These dietary and feeding-related data may be used as a covariate for the gut microbiome analysis, as gut composition is highly diet dependent.

#### Concomitant Treatments and Medications

Trial infants may receive any medically needed treatment, therapy, or medication, and there are no unauthorized treatments or medications. Parents or LARs will be requested not to give any prebiotic, or probiotic supplement from enrollment until after the stool sample is collected, just before V5 (age 12 months), unless medically prescribed or recommended by the infant’s physician. If given, prebiotic or probiotic supplements will be considered as concomitant medications and will be reported in the electronic case report form (eCRF) until V5.

#### Concomitant Diets

Introduction of complementary foods before V2 (infant age of 3 months) will be considered unauthorized per current recommendations for feeding healthy newborn infants and recorded as nontrial feeding in the eCRF. Additionally, any formula other than the trial formulas (CG and EG infants only) from the collection of the 1st stool sample (home [H1]) until intervention end (V3), and any infant formula in the BG before V2 will also be recorded as nontrial feeding in the eCRF. Finally, any prebiotic- or probiotic-containing foods until V2 will be reported as nontrial feeding in the eCRF for any of the groups.

### Data Analysis

#### Sample Size

The sample size was calculated based on the primary end point of the trial and an additional nonrandomized breastfed arm. To demonstrate a statistically significant increase of 8% in relative *Bifidobacteria* abundance at 3 months in the EG versus CG infants with a power of 80% and an α-level of 5%, a sample size of 105 per arm (including a 20% attrition rate) will be needed. For the nonrandomized breastfed reference arm, assuming a noninferiority margin of 20% in *Bifidobacteria* abundance with the experimental formula arm, the required sample size will be 52 infants (including a 20% dropout rate). Therefore, the total sample needed for this trial will be 262 infants (105 EG, 105 CG, and 52 BG).

#### Statistical Analysis

A staged statistical analysis (SSA) is defined as any statistical analysis performed when recruitment is completed, all participants have completed a predefined specific time point before the last participant last visit has been performed, and before the final database for the trial is locked.

The estimates computed at the SSA are considered final as all the participants have passed the predefined specific time point. The SSA will not lead to any modification in the design and subsequent conduct of the trial. Its sole purpose is to accelerate the availability of the key end point estimates. For this study, in addition to the final analysis, 2-stage statistical analyses are planned at 3- and 6-month visits. The analysis of the primary end point will be included in the first SSA.

Data will be analyzed on the following analysis populations:

1. Full analysis set (FAS): the FAS will exclude randomized participants who never took any of the assigned product (trial formula or breastmilk), participants who failed to satisfy trial entry eligibility criteria, or had no postrandomization data. This will be derived according to the assigned product group per randomization.
2. Safety analysis set (SAF): the SAF will include all participants in the FAS dataset with documented use of at least one feeding of the trial formula or breastmilk for the BG, classified according to the feeding actually received, irrespective of the randomization assignment.
3. Per protocol set (PPS): the PPS will include participants in the FAS with no major protocol deviations. Participants with major protocol deviations related to Good Clinical Practice (eg, ICF process not followed) will not be removed from PPS by default. A separate listing of these participants will be provided in the statistical report. The protocol deviations (eg, product compliance or visit window) will be defined before trial start. The listing of the protocol deviations will be reviewed during the blinded data review meeting.

The primary end point will be assessed on both FAS and PPS populations. All secondary end points will be analyzed on the FAS except for safety end points, which will be on the SAF. No interim analyses are planned for this study.

For all categorical and continuous variables, appropriate descriptive statistics will be calculated by visit, feeding groups, and delivery mode. The primary end point of the difference in *Bifidobacteria* abundance between EG and CG infants will be analyzed using analysis of covariance (ANCOVA), correcting for baseline, site, gender, and mode of delivery. If the investigation of data reveals an inflation of the null values (no detected *Bifidobacteria* in samples), then the ANCOVA model will be replaced by a 0-inflated mixture model. For continuous secondary end points, ANCOVA models correcting for baseline, site, gender, and delivery will be used for analyses. For categorical secondary end points, logistic regression models will be used to compare between feeding groups at each time point separately. Fecal metabolism and immune health markers will be analyzed according to dry stool weight, using an ANCOVA model at each time point to compare between feeding groups, correcting for baseline, site, gender, and mode of delivery. The necessity to log-transform the end points or to use nonparametric methods will be assessed during the blind data review and addressed in the statistical analysis plan. During the final analysis, the models may be modified to a mixed model repeated measurements to consider the longitudinal nature of the data. AEs will be compared between feeding groups using the Fisher exact test.

Missing data will be assumed to be missing at random unless the blind review of the data demonstrates a pattern of missingness, in which case, multiple imputation for the primary outcome of *Bifidobacteria* abundance at 3 months may be used. In the case of outliers for any end point, robust approaches or sensitivity analyses may be used. Statistical testing for all end points will be 2-sided and at a 5% significance level. All statistical analyses will be performed in SAS Life Science Analytics Framework (version 5.4.1; SAS Institute Inc).

#### Compliance With Investigational Product

Compliance with feeding interventions will be monitored throughout the trial using various tools, such as a formula milk intake diary and breastfeeding questionnaire at specified visits. Data on nontrial feeding will also be recorded at specific time points to inquire about regimen interruptions and consumption of unauthorized feedings or supplements. Each trial day in which trial formula intake or exclusive breastmilk feedings are interrupted will be counted as a noncompliant day. Throughout the trial, noncompliance will be calculated based on formula-fed infants being off trial formula or breastfed infants being off breast milk and consumption of prebiotics or probiotic-containing supplements, with some minor modifications based on specific visits.

The number of noncompliant and compliant days will be determined, and a compliance rate as a percentage of compliant days concerning total potential days of exclusive feeding (approximately 100 days, but individual for each infant, depending on the enrollment age) will be calculated for each participant and dropout participant. Participants with a compliance rate of less than 80% will be considered as having a major protocol deviation and will not be included in the PPS. All noncompliance between V2 and V5 will be considered minor protocol deviations.

#### Trial Discontinuation

Discontinuation from the trial product may happen for the following reasons: parents or LARs withdraw consent to participate, or the investigator decides to discontinue the trial if they opine that continued exposure to the trial product would be detrimental to the participant’s well-being. No additional data will be collected, and no further analyses will be performed if consent is withdrawn. The reason for premature discontinuation will be documented, and the investigator will follow up regarding any unresolved AEs.

### Data Management, Monitoring, and Dissemination

#### Data Management

Site personnel will manually enter source data (clinical findings and observations, laboratory and test data, questionnaires, site notebooks, and paper diaries) into the electronic Case Report Form (eCRF) web database, which is 21 CFR Part 11 compliant. The clinical data manager will review and validate all entered data. The eCRF database will be locked after cleaning of the data, which includes review, query resolution, signatures by the investigators, and determination that the database is ready for analysis. All documents on the conduct of the study will be kept by the investigator for 5 years after the study’s completion.

#### Data Monitoring

The monitoring plan will be developed and agreed upon between the sponsor and the contract research organization responsible for the monitoring at the site on the monitoring activities to be compliant with the protocol, Good Clinical Practice Guidelines, local regulations, and the sponsor’s procedures, and to ensure the subjects’ well-being and data integrity. At agreed-upon times during the trial and after the trial has been completed, the investigator will allow sponsor representatives to periodically review the eCRF and corresponding records (source documents) of each trial participant. These monitoring visits will allow the sponsor or sponsor representative to examine trial progress, verify completeness and accuracy of eCRF, resolve any inconsistencies in the records, and ensure that all protocol requirements and obligations are being fulfilled. The sponsor representatives or external auditors will be permitted by the investigator to conduct an audit of the trial during the trial or after the trial ends. This will be to ensure compliance with the Good Clinical Practice Guidelines, local regulations, the trial protocol, and the sponsor’s procedures, as well as evaluating the accuracy of the trial data.

Regular data quality reviews are organized to identify any data inconsistencies. To ensure data quality, automatic edit checks that identify inconsistencies have been set up and flagged as queries during the data entry process. For more complex data where these automated checks cannot be implemented, manual review will be performed.

### Ethical Considerations

Ethics approvals were granted by all the independent ethics committees of all the hospitals joining in the study, which are the Ethics Committee of Xinhua Hospital Affiliated to Shanghai Jiao Tong University School of Medicine (XHEC-C-2024-024-2), the Ethics Committee of Shanghai First Maternal and Infant Hospital (2024 027), the Ethics Committee of The International Peace Maternity and Child Health Hospital of China Welfare Institute (GKLW-A-2024-024-02), the Ethics Committee of the Peking University Third Hospital (2024 878-02), and the Ethics Committee of Nanjing Women and Children’s Healthcare Hospital (2024FYYL007). All parents or LARs will sign an informed consent form (see example in Additional File S2 in Multimedia Appendix 1). In the event of a screen failure, that is, consent was signed but the participant was not subsequently enrolled, information related to the reason for screen failures will be documented.

This study will be conducted per the ethical principles outlined in the Declaration of Helsinki and in compliance with all applicable regulatory requirements, including China’s Clinical Trial laws, the Personal Information Protection Law, and the Measures for Science and Technology Ethics Reviews (effective as of December 2023).

All participants will receive comprehensive written and verbal information about this study’s objectives, procedures, potential risks, and benefits. Written informed consent will be obtained from each participant before any study-related procedures.

To ensure the protection of participants’ rights, safety, and well-being:

- AEs will be monitored and managed per regulatory and ethical standards.
- Personal and health data will be collected, stored, and processed in full compliance with Personal Information Protection Law requirements.
- Participants will be informed of their right to withdraw from the study at any time without penalty.

Special attention will be given to the recruitment of breastfeeding mothers. Per the WHO International Code of Marketing of Breast-Milk Substitutes, no coercive or promotional practices were used. Participation will be entirely voluntary, and all information will be provided neutrally and respectfully to ensure informed and independent decision-making.

The study will be conducted by qualified investigators and supported by institutional quality assurance processes to ensure ethical conduct and regulatory compliance throughout. The study has been registered on ClinicalTrials.gov (NCT06361719), and results will be submitted to a peer-reviewed journal for dissemination; the role of authors and contributors will be defined according to International Committee of Medical Journal Editors guidelines. The study will be carried out according to the SPIRIT (Standard Protocol Items: Recommendations for Interventional Trials) guidelines. The completed SPIRIT checklist is provided in Additional File S3 in Multimedia Appendix 1.

Every precaution will be taken to protect the privacy of research participants and the confidentiality of their personal information. The investigator will ensure protection of the participant’s personal data and of all reports, publications, participant samples, and any other disclosures, except where required by law. Participants are identified only by a participant identification number and site identification number to maintain participant confidentiality. All participant study records will be kept safely in an access-controlled area. Identification code lists linking participant names to participant identification numbers will be stored separately from participant records. In case of data transfer, the sponsor will maintain high standards of confidentiality and protection of participant personal data. Clinical personal information will not be released without the written permission of the participant, except for monitoring by regulatory authorities or the study sponsor. The investigator or designee will comply with all applicable data protection laws and implement and maintain appropriate technical and organizational measures against the unauthorized or unlawful acquisition, access to, or processing of Personal Data and against the accidental loss, disclosure, or destruction of, or damage to, Personal Data.

## Results

The study was initiated in July 2024, with the first participant’s first visit at the end of July 2024. As of June 2025, a total of 122 participants were recruited, and 74 completed visit 2 (3-month time point). The study is expected to be completed (the last participant’s last visit) by the first half of 2026. All data will be analyzed at the end of the study (after database lock, to maintain the double-blind nature of the study), followed by publications in peer-reviewed journals.

## Discussion

The Starter Liquid Infant Formula Trial (STARLIT) is among the first few clinical trials that will investigate the efficacy of the 2 HMOs, 2-fucosyllactose and lacto-N-neotetraose, on gut health, gut barrier integrity, and immune response, together with clinically relevant outcomes such as illness, infections, growth, and quality of life among Chinese infants born at term. *Bifidobacteria*, a key microbiome taxon during early life development, are often depleted in the gut of an infant receiving infant formula compared to breastfeeding [43]. One of the main drivers of this difference is the absence of HMOs in infant formula compared with breastmilk [44]. We therefore anticipate that consumption of an infant containing the 2 selected HMOs formula from birth to age 6 months will favor the growth of gut *Bifidobacteria* compared with the CG and should bring *Bifidobacteria* levels closer to those in the breastfeeding arm of the study. Given the central role of *Bifidobacteria* in gut microbiome maturation, we expect that other microbial taxa and functions may also be affected by the presence of the 2 HMOs and contribute to directing the gut microbiome maturation closer to the 1 observed in breastfed infants [45]. Finally, both *Bifidobacteria* and HMOs have been linked to multiple health outcomes [32]. With the breadth of end points collected in this study, we hope to be able to explore the importance of the addition of HMOs in early-life infant formula beyond the gut microbiome. Breastfeeding is the reference for infant nutrition as it is the best way to support optimal growth and development. Providing suitable solutions for infants that cannot be breastfed and that mimic breast milk composition as best as possible has become possible with ongoing research on breast milk composition as well as technological progress. To date, differences in health outcomes between breastfed and formula-fed infants remain, which may be attributed to the absence of key bioactive compounds such as HMOs in standard infant formulas [46,47]. HMOs are the third largest solid component in human milk, following lactose and lipids. They offer a wide range of functional benefits, including supporting immune and cognitive development [1,3,32,33,48]. These benefits may mostly be mediated by the impact of HMOs on the gut microbiome and its metabolic activity [49,50]. HMOs are nondigestible oligosaccharides that reach the lower part of the infant digestive track, where they can be used and transformed by the maturing gut microbiome. Specifically, they support the growth and metabolic activity of *Bifidobacteria* in early stages of life, which has been associated with multiple health benefits [51]. Technological and regulatory advancements now allow the inclusion of oligosaccharides identical to those found in human milk, such as 2-fucosyllactose and lacto-N-neotetraose, in infant formulas in China [32,33]. Hence, generating clinical evidence that supports the efficacy of these HMOs in this population is much warranted.

We hypothesize that the addition of 2-fucosyllactose and lacto-N-neotetraose to the infant formula will promote the growth of beneficial *Bifidobacteria* and drive the gut microbiome of infants closer to that of breastfed infants. Additionally, we propose that this bifidogenic effect may further benefit infant gut and immune health. Previous studies have shown that the fecal microbiome of most infants from China falls within a distinct enterotype from the rest of the world. More specifically, this enterotype found most predominantly in China is characterized by a relatively high abundance of *Proteobacteria* and more specifically of Enterobacteriaceae [52]. These taxa are considered opportunistic pathogens with their ability to alter the composition of the gut microbiome and favor gut dysbiosis [53,54]. They also have proinflammatory properties and have been associated with IBD pathogenesis and progression [55,56]. In contrast, among infants from the West, *Bifidobacterium* was found to be the dominant genus of the intestinal microbiota. Decline in *Bifidobacteria* and gut microbial dysbiosis has been linked to the increasing incidence of allergies and autoimmune diseases [57,58]. As mentioned earlier, in infants given formula supplemented with 2-fucosyllactose and lacto-N-neotetraose compared to control, there were significantly fewer reported incidences of bronchitis, respiratory tract infections, and use of antibiotics [27,30]. This observation has been linked not only to the bifidogenic effects of these HMOs but also to *Bifidobacterium*-related metabolic changes in the gut environment [28,29,59]. Hence, characterizing the role of HMOs in modulating the gut microbiome of Chinese infants and, in turn, assessing its impact on the immune health is central to this study [27-30,57-61].

Our study has several strengths. Its double-blinded design will ensure blinding of the participants, investigators, data collectors, outcome assessors, and data analysts to minimize bias in the reporting of the study outcomes. The study will be conducted across 5 different sites representing a wide geography within China and enabling a nonpreceded characterization of the effects of HMO supplementation in this population. Regarding the intervention products, the administration of a control formula identical to the test formula, apart from its HMO content, would allow us to clarify the efficacy of supplementation by 2-fucosyllactose and lacto-N-neotetraose with no further nutritional confounding factors. Yet, the 2 formulas must be precisely fully compliant with infant nutritional needs at this age. The nonrandomized BG will serve as a reference and help to gain insights into the contribution of these HMOs in shifting the gut microbiome and all other developmental outcomes closer to those of breastfed infants. The benefit of breastfeeding will be discussed with all parents who will meet a qualified lactation consultant before enrollment to ensure that they make an informed decision that is best for their infant. The service of a lactation consultant will be available over the course of the study to allow any parent to switch to breastfeeding at any time point.

The prospective and longitudinal nature of this study will allow us to collect detailed information on a comprehensive set of outcomes during the first year of life. Importantly, the fecal microbiome will be profiled using the most advanced and standard methodologies, such as NGS, in addition to quantitative polymerase chain reaction. This should enable us to shed light on the effects of HMOs on the dynamic maturation of the gut microbiome of Chinese infants at the taxonomic but also functional levels. HMO-mediated microbiome modulation of immune system development and fitness will be carefully monitored. We will capture infant illness weekly for 1 year using an IID, providing great granularity over AE occurrence. These IIDs will be used to generate an automated diagnosis that will be reviewed and confirmed by a study physician. Additionally, for the first time, we will use a novel Pediatric Immune System Index to assess immune competence that will allow the detailed characterization of gastrointestinal, upper respiratory tract infection, LRTI, and skin allergies by recording the duration, number of episodes, severity of health care visits, and treatment received. To complement this, fecal immune markers will also be quantified. Furthermore, we will be using validated questionnaires to capture details about gastrointestinal symptoms, sleep patterns, and quality of life to enhance our understanding of the effect of HMOs and their microbiome-modulating properties on infants’ well-being [27]. Importantly, we will also collect covariates, including maternal education, delivery mode, family history of allergy, infant dietary intake, and infant feeding (breastfeeding and formula feeding), medications, and treatments to adjust our analysis for potential confounding effects. In our study, the randomization is stratified by delivery mode. We hope to gain important insights into the effect of HMO supplementation, particularly among infants born by CS, in whom lower colonization rates of beneficial bacteria such as *Bifidobacterium* and *Bacteroides*, and higher colonization rates of *Clostridium*, *Lactobacillus*, *Enterobacter*, *Enterococcus*, and *Staphylococcus* have been reported [62]. This is particularly relevant considering the high rates of elective CS in China with an increase reported from 28.8% in 2008 to 36.7% in 2018 [63] and reaching up to 43.79% in 2019 according to the data published by the National Health Service and Quality Safety Report (obstetrics) in 2020 [64] This is far above the CS rates of 10%-15% of all deliveries suggested as reasonable by the WHO [65,66] and likely impacts the immune development of their infants, risk for allergy, atopy, and asthma, and intestinal gut microbiome diversity [67].

We acknowledge a few limitations in our study. While the study uses a comprehensive set of tools for assessing several outcomes, these may be perceived as burdensome to some subjects. Hence, the choice of not including systemic immune markers in addition to feces to reduce burden and risk linked to invasive procedures was made. Finally, generalization of results outside of China may be limited due to the geographical specificity of their gut microbiome.

In conclusion, this double-blind randomized controlled trial with a nonrandomized BG will be among the first studies in China to investigate the efficacy of a ready-to-feed infant formula containing 2-fucosyllactose and lacto-N-neotetraose in a matrix containing a unique whey protein concentrate coenriched in α-lactalbumin and MFGM on gut and immune health, as well as the effects on tolerance, growth, sleep, and quality of life. The much-anticipated results from this trial will strengthen the evidence base regarding the beneficial effects of HMOs in Chinese infants, as the study design should enable us to demonstrate that the addition of HMOs in infant formula contributes to supporting Bifidobacterium growth in the infant gut as well as the appropriate maturation of the gut microbiome and immune functions. This may help drive recommendations on target doses that are appropriate for observing beneficial changes in gut microbiome, gut maturation, gut barrier integrity, immune response, and other developmental outcomes.

## Appendix

Multimedia Appendix 1 Additional file S1: plans for collection, laboratory evaluation, and storage of biological specimens for genetic or molecular analysis in the current trial and for future use in ancillary studies. Additional file S2: model consent form and other related documentation given to participants and authorized surrogates. Additional file S3: SPIRIT checklist.

## Abbreviations

AE: adverse event
ANCOVA: analysis of covariance
APGAR: appearance, pulse, grimace, activity, and respiration
BG: breastfed group
CG: control group
CP: comparator product
CS: cesarean section
eCRF: electronic case report form
EG: experimental group
FAS: full analysis set
HMO: human milk oligosaccharide
IID: infant illness diary
LAR: legally authorized representative
LRTI: lower respiratory tract infection
MFGM: milk-fat-globule membrane
NGS: next-generation sequencing
PPS: per protocol set
SAF: safety analysis set
SPIRIT: Standard Protocol Items: Recommendations for Interventional Trials
SSA: staged statistical analysis
WHO: World Health Organization

## Data Availability

Data sharing is not applicable to this paper, as this is a protocol and no datasets were generated or analyzed during this study.
