# Supplementary material for: Efficacy of a Ready-to-Feed Starter Liquid Infant Formula Containing 2-Fucosyllactose and Lacto-N-Neotetraose in Chinese Infants: Protocol for a Double Blind, Randomized Controlled Trial
Source: JMIR Res Protoc. 2025 Oct 31;14:e66489. doi: 10.2196/66489 (PMC12619021; doi:10.2196/66489)
Supplement: Multimedia Appendix 1 [file resprot_v14i1e66489_app1.pdf]

Additional files for: **Efficacy of a ready to feed starter liquid infant formula containing 2'FL and LNnT in Chinese infants: "STARLIT" trial** (**STAR**ter **LI**quid infant formula **T**rial with 2'FL and LNnT in Chinese infants: a double-blind, randomized controlled trial with a breastfed reference group)

**Additional File 1.** Plans for collection, laboratory evaluation, and storage of biological specimens for genetic or molecular analysis in the current trial and for future use in ancillary studies.

**Additional File 2.** Model consent form and other related documentation given to participants and authorized surrogates.

**Additional File 3.** Standard Protocol Items: Recommendations for Interventional Trials (SPIRIT) checklist.

**Additional File 1:** Plans for collection, laboratory evaluation, and storage of biological specimens for genetic or molecular analysis in the current trial and for future use in ancillary studies.

In this study, the only biological specimen collected will be the fecal samples of the infant. In total 5 fecal samples will be collected per participant. Collection will happen on average 3 months apart at baseline and few days prior to V2, V3, V4, and V5.

**Collection**

Fecal samples will be collected at home by parents/LARs who will be provided with an instruction booklet and collection kits for each sampling occasion by the investigators at site at V1. For sampling, parents will be asked to transfer fecal material from their infant diaper into two separate tubes provided within the dedicated kit that will be coded according to subject ID.

**Storage**

Parents will be asked to store the samples frozen until arrangement is made to ship the samples on ice to the study site. Transfer should occur within a window of no more than 3 days after fecal sample collection. Transfer of samples between parent’s home and the study site should be done in a cooling bag provided at the beginning of the study to avoid samples thawing. Upon arrival at study site, samples will be transferred to a -80°C freezer. If at any point samples are to be transferred to another storage or analysis site, those will be transported on dry ice and stored at -80°C upon arrival. All received samples are logged and tracked into a system following good laboratory practice (GLP) standards upon destruction at the latest 5 years after study end.

**Laboratory evaluation**

Fecal samples will be used for microbiome and metabolome profiling as well as quantification of immune markers.

*Fecal microbiome profiling*

DNA will be extracted from the fecal samples and microbiome composition (at taxonomic and functional level) will be assessed using next generation sequencing methodology. This will be

conducted by certified and qualified external laboratories contracted by the sponsor. In addition, DNA aliquot will be used to perform qPCR analysis for quantification of *Bifidobacterium*.

*Fecal metabolome profiling*

Fecal water will be extracted from the samples and then analyses using mass spectrometry or magnetic nuclear resonance (NMR) methodology for the targeted and untargeted metabolomic profiling. Fecal organic acid measurement will be performed on the same aliquot *via* HPLC and pH through a standard pH meter.

*Fecal immune markers*

Fecal immune markers, namely secretory IgA (sIgA), lipocalin-2, calprotectin, α-1-antitrypsin will be assessed by ELISA.

**Further use**

Not applicable.

**Additional File 2:** Model consent form and other related documentation given to participants and authorized surrogates.

At screening visit (V1), parents/LARs will be explained and given an informed consent form (ICF) for the study and an ICF for Personal Information Protection Law (PIPL) preliminarily approved by the relevant ethical committee and applicable authorities. They will sign the 2 consent forms prior their infant takes part to the study. Key information contained in these documents are described thereafter.

**ICF**

The ICF must be signed and dated by at least one of the parents/ legally accepted representatives (LARs) as well as the investigator, that any infant identified as suitable for the study may take part and that subsequent data may be used. In total 3 copies must be signed, of which 2 will be kept by parents/ LARs and 1 by the site in the investigator file for a duration of 5 years in accordance with local regulations in China. The ICF provides a complete description of the study including key point of contact; the purpose of the study; the product that will be tested; implication of taking part to the study; description of the study procedures (that may differ according to the study arm – formula fed or breast fed); possible benefits and risks. It also encompasses standard study information such as title, investigator name and institution, study participant name and subject code (to be filled in by the investigator after signature) Information related to potential costs and payment associated with the study and where they may be able to find more information is also provided. All those points will also be described in detail by the study team at V1 prior to signing, giving also the opportunity to the parents to ask question and request clarification.

After sufficient time, parents are asked if they agree to the following and sign that their infant can participate to the study:

- They have been told everything they wanted to know about the study and have understood the spoken and written instructions provided.
- They have been given time and the chance to look at the information described above.
- The agreement for their infant to take part is given of their own free will.

- They will inform the study doctor if they decide to withdraw their infant's participation from the study.
- They understand that a liability insurance has been taken according to the laws and regulations of China.
- The Sponsor representatives, ethical committee/institutional board or any other competent authorities may look at their infant's personal records.
- They understand that in case of special situations like sanitary conditions not allowing them to go on site, the study visits might be done at home or remotely (by phone).
- They know that their infant's coded health-related personal data and biological materials may be transferred to third parties in Switzerland and/or abroad for research purposes.
- They release the investigator of his/her professional secrecy obligations to the extent necessary for such transfer to third parties.

**ICF for PIPL**

This document refers to following the Chinese regulation, called PERSONAL INFORMATION PROTECTION LAW (EFFECTIVE DATE: 1 NOVEMBER 2021, hereinafter referred to as "PIPL"), this letter clarifies how personal information collected for this study are protected and what are the rights of the participants and their parents/ LARs. This form must be signed and dated by the parents/ LARs and the investigator. In total 3 copies must be signed by all parties, 2 of which are kept by parents/ LARs and one by the site in the investigator file for 5 years as per local regulations.

More specifically, the form contains information on: the processors involved in the study; the kind of personal information collected; data confidentiality and protection, type of processing; data sharing outside of China; results disclosure; parent's rights regarding their infant's personal information and procedures to exercise their rights to withdraw, file a complaint or make rectifications.

Participants are then informed of the following before the signature page: 'By signing the ICF, you are giving your explicit consent to relevant parties for processing your personal data to the extent permitted by PIPL for and within the aforementioned purposes'.

**Additional File 3.** Standard Protocol Items: Recommendations for Interventional Trials (SPIRIT) Checklist.

| Section/item | Item No | Description | Addressed on page number |
|---|---|---|---|
| **Administrative information** | | | |
| Title | 1 | Descriptive title identifying the study design, population, interventions, and, if applicable, trial acronym | p.1 |
| Trial registration | 2a | Trial identifier and registry name. If not yet registered, name of intended registry | p.2 |
| | 2b | All items from the World Health Organization Trial Registration Data Set | All text |
| Protocol version | 3 | Date and version identifier | p.4 |
| Funding | 4 | Sources and types of financial, material, and other support | p.2 |
| Roles and responsibilities | 5a | Names, affiliations, and roles of protocol contributors | pp.1-2 |
| | 5b | Name and contact information for the trial sponsor | p.2 |
| | 5c | Role of study sponsor and funders, if any, in study design; collection, management, analysis, and interpretation of data; writing of the report; and the decision to submit the report for publication, including whether they will have ultimate authority over any of these activities | p.2 |
| | 5d | Composition, roles, and responsibilities of the coordinating centre, steering committee, endpoint adjudication committee, data management team, and other individuals or groups overseeing the trial, if applicable (see Item 21a for data monitoring committee) | pp.2, 24-26 |
| **Introduction** | | | |
| Background and rationale | 6a | Description of research question and justification for undertaking the trial, including summary of relevant studies (published and unpublished) examining benefits and harms for each intervention | pp.6-7 |
| | 6b | Explanation for choice of comparators | pp.6-7 |

| | | | |
|---|---|---|---|
| Objectives | 7 | Specific objectives or hypotheses | p.8 |
| Trial design | 8 | Description of trial design including type of trial (eg, parallel group, crossover, factorial, single group), allocation ratio, and framework (eg, superiority, equivalence, noninferiority, exploratory) | p.8; Figure 1 |
| **Methods: Participants, interventions, and outcomes** | | | |
| Study setting | 9 | Description of study settings (eg, community clinic, academic hospital) and list of countries where data will be collected. Reference to where list of study sites can be obtained | pp.11 |
| Eligibility criteria | 10 | Inclusion and exclusion criteria for participants. If applicable, eligibility criteria for study centres and individuals who will perform the interventions (eg, surgeons, psychotherapists) | pp.10-11 |
| Interventions | 11a | Interventions for each group with sufficient detail to allow replication, including how and when they will be administered | pp.13-14 |
| | 11b | Criteria for discontinuing or modifying allocated interventions for a given trial participant (eg, drug dose change in response to harms, participant request, or improving/worsening disease) | pp.14, 24 |
| | 11c | Strategies to improve adherence to intervention protocols, and any procedures for monitoring adherence (eg, drug tablet return, laboratory tests) | pp.23-25 |
| | 11d | Relevant concomitant care and interventions that are permitted or prohibited during the trial | pp.19-20 |
| Outcomes | 12 | Primary, secondary, and other outcomes, including the specific measurement variable (eg, systolic blood pressure), analysis metric (eg, change from baseline, final value, time to event), method of aggregation (eg, median, proportion), and time point for each outcome. Explanation of the clinical relevance of chosen efficacy and harm outcomes is strongly recommended | pp.14-20 |
| Participant timeline | 13 | Time schedule of enrolment, interventions (including any run-ins and washouts), assessments, and visits for participants. A schematic diagram is highly recommended (see Figure) | Figure 1, Table 1, Text |
| Sample size | 14 | Estimated number of participants needed to achieve study objectives and how it was determined, including clinical and statistical assumptions supporting any sample size calculations | pp.20-21 |
| Recruitment | 15 | Strategies for achieving adequate participant enrollment to reach target sample size | p.12 |

**Methods: Assignment of interventions (for controlled trials)**

Allocation:

| | | | |
|---|---|---|---|
| Sequence generation | 16a | Method of generating the allocation sequence (eg, computer-generated random numbers), and list of any factors for stratification. To reduce predictability of a random sequence, details of any planned restriction (eg, blocking) should be provided in a separate document that is unavailable to those who enroll participants or assign interventions | pp.12-13 |
| Allocation concealment mechanism | 16b | Mechanism of implementing the allocation sequence (eg, central telephone; sequentially numbered, opaque, sealed envelopes), describing any steps to conceal the sequence until interventions are assigned | pp.12-13 |
| Implementation | 16c | Who will generate the allocation sequence, who will enroll participants, and who will assign participants to interventions | pp.12-13 |
| Blinding (masking) | 17a | Who will be blinded after assignment to interventions (eg, trial participants, care providers, outcome assessors, data analysts), and how | pp.12-13 |
| | 17b | If blinded, circumstances under which unblinding is permissible, and procedure for revealing a participant's allocated intervention during the trial | pp.12-13 |

**Methods: Data collection, management, and analysis**

| | | | |
|---|---|---|---|
| Data collection methods | 18a | Plans for assessment and collection of outcome, baseline, and other trial data, including any related processes to promote data quality (eg, duplicate measurements, training of assessors) and a description of study instruments (eg, questionnaires, laboratory tests) along with their reliability and validity, if known. Reference to where data collection forms can be found, if not in the protocol | pp.14-20 |
| | 18b | Plans to promote participant retention and complete follow-up, including list of any outcome data to be collected for participants who discontinue or deviate from intervention protocols | pp.14-20 |
| Data management | 19 | Plans for data entry, coding, security, and storage, including any related processes to promote data quality (eg, double data entry; range checks for data values). Reference to where details of data management procedures can be found, if not in the protocol | pp.24-26 |

| | | | |
|---|---|---|---|
| Statistical methods | 20a | Statistical methods for analysing primary and secondary outcomes. Reference to where other details of the statistical analysis plan can be found, if not in the protocol | pp.21-23 |
| | 20b | Methods for any additional analyses (eg, subgroup and adjusted analyses) | pp.21-23 |
| | 20c | Definition of analysis population relating to protocol non-adherence (eg, as randomised analysis), and any statistical methods to handle missing data (eg, multiple imputation) | pp.21-23 |
| **Methods: Monitoring** | | | |
| Data monitoring | 21a | Composition of data monitoring committee (DMC); summary of its role and reporting structure; statement of whether it is independent from the sponsor and competing interests; and reference to where further details about its charter can be found, if not in the protocol. Alternatively, an explanation of why a DMC is not needed | pp.24-25 |
| | 21b | Description of any interim analyses and stopping guidelines, including who will have access to these interim results and make the final decision to terminate the trial | pp.22 |
| Harms | 22 | Plans for collecting, assessing, reporting, and managing solicited and spontaneously reported adverse events and other unintended effects of trial interventions or trial conduct | p.18 |
| Auditing | 23 | Frequency and procedures for auditing trial conduct, if any, and whether the process will be independent from investigators and the sponsor | p.25 |
| **Ethics and dissemination** | | | |
| Research ethics approval | 24 | Plans for seeking research ethics committee/institutional review board (REC/IRB) approval | p.26 |
| Protocol amendments | 25 | Plans for communicating important protocol modifications (eg, changes to eligibility criteria, outcomes, analyses) to relevant parties (eg, investigators, REC/IRBs, trial participants, trial registries, journals, regulators) | p.26 |
| Consent or assent | 26a | Who will obtain informed consent or assent from potential trial participants or authorised surrogates, and how (see Item 32) | p.26 |
| | 26b | Additional consent provisions for collection and use of participant data and biological specimens in ancillary studies, if applicable | p.26 |

| | | | |
|---|---|---|---|
| Confidentiality | 27 | How personal information about potential and enrolled participants will be collected, shared, and maintained in order to protect confidentiality before, during, and after the trial | pp.25-26 |
| Declaration of interests | 28 | Financial and other competing interests for principal investigators for the overall trial and each study site | p.2 |
| Access to data | 29 | Statement of who will have access to the final trial dataset, and disclosure of contractual agreements that limit such access for investigators | p.24 |
| Ancillary and post-trial care | 30 | Provisions, if any, for ancillary and post-trial care, and for compensation to those who suffer harm from trial participation | Not applicable |
| Dissemination policy | 31a | Plans for investigators and sponsor to communicate trial results to participants, healthcare professionals, the public, and other relevant groups (eg, via publication, reporting in results databases, or other data sharing arrangements), including any publication restrictions | p.26 |
| | 31b | Authorship eligibility guidelines and any intended use of professional writers | pp.26 |
| | 31c | Plans, if any, for granting public access to the full protocol, participant-level dataset, and statistical code | p.2 |
| **Appendices** | | | |
| Informed consent materials | 32 | Model consent form and other related documentation given to participants and authorised surrogates | Additional File 2 |
| Biological specimens | 33 | Plans for collection, laboratory evaluation, and storage of biological specimens for genetic or molecular analysis in the current trial and for future use in ancillary studies, if applicable | Additional File 1 |

The SPIRIT checklist is copyrighted by the SPIRIT Group under the Creative Commons "Attribution-NonCommercial-NoDerivs 3.0 Unported" license.
